# Nasal Carriage of *Staphylococcus aureus* among Children in the Ashanti Region of Ghana

**DOI:** 10.1371/journal.pone.0170320

**Published:** 2017-01-20

**Authors:** Daniel Eibach, Michael Nagel, Benedikt Hogan, Clinton Azuure, Ralf Krumkamp, Denise Dekker, Mike Gajdiss, Melanie Brunke, Nimako Sarpong, Ellis Owusu-Dabo, Jürgen May

**Affiliations:** 1 Infectious Disease Epidemiology, Bernhard Nocht Institute for Tropical Medicine, Hamburg, Germany; 2 Deutsche Gesellschaft für Internationale Zusammenarbeit (GIZ), Bonn, Germany; 3 German Centre for Infection Research (DZIF), Hamburg-Borstel-Lübeck, Germany; 4 Kumasi Centre for Collaborative Research in Tropical Medicine (KCCR), Kumasi, Ghana; 5 Institute of Medical Microbiology, Immunology and Parasitology, University Clinic of Bonn, Bonn, Germany; 6 Institute for Pharmaceutical Microbiology, University of Bonn, Bonn, Germany; Rockefeller University, UNITED STATES

## Abstract

**Background:**

Nasal carriage with *Staphylococcus aureus* is a common risk factor for invasive infections, indicating the necessity to monitor prevalent strains, particularly in the vulnerable paediatric population. This surveillance study aims to identify carriage rates, subtypes, antimicrobial susceptibilities and virulence markers of nasal *S*. *aureus* isolates collected from children living in the Ashanti region of Ghana.

**Methods:**

Nasal swabs were obtained from children < 15 years of age on admission to the Agogo Presbyterian Hospital between April 2014 and January 2015. *S*. *aureus* isolates were characterized by their antimicrobial susceptibility, the presence of genes encoding for Panton-Valentine leukocidin (PVL) and toxic shock syndrome toxin-1 (TSST-1) and further differentiated by *spa*-typing and multi-locus-sequence-typing.

**Results:**

Out of 544 children 120 (22.1%) were colonized with *S*. *aureus*, with highest carriage rates during the rainy seasons (27.2%; p = 0.007), in females aged 6–8 years (43.7%) and males aged 8–10 years (35.2%). The 123 isolates belonged to 35 different *spa*-types and 19 sequence types (ST) with the three most prevalent *spa*-types being t355 (n = 25), t84 (n = 18), t939 (n = 13), corresponding to ST152, ST15 and ST45. Two (2%) isolates were methicillin-resistant *S*. *aureus* (MRSA), classified as t1096 (ST152) and t4454 (ST45), and 16 (13%) were resistant to three or more different antimicrobial classes. PVL and TSST-1 were detected in 71 (58%) and 17 (14%) isolates respectively.

**Conclusion:**

*S*. *aureus* carriage among Ghanaian children seems to depend on age, sex and seasonality. While MRSA rates are low, the high prevalence of PVL is of serious concern as these strains might serve not only as a source for severe invasive infections but may also transfer genes, leading to highly virulent MRSA clones.

## Background

*Staphylococcus aureus* contributes significantly to morbidity and mortality worldwide, causing a broad spectrum of diseases [1,2]. *S*. *aureus* nasal colonization has been identified as the most important risk factor for subsequent invasive infections [3]. An estimated 30% of humans are nasal carriers of *S*. *aureus* [4], however carriage rates vary with geographic location, seasonality, age and sex [1]. Studies among Dutch children revealed a decreasing carriage rate during the first year of life, remaining stable at 20–30% until it increases again to 40–50% between the age of 6 to 12 years [5,6]. In West Africa those rates might be considerably different due to co-colonization with other pathogens or particular living conditions, such as large family sizes and lower sanitary standards, which are all associated with *S*. *aureus* nasal carriage [7,8]. Studies from West and Central Africa show carriage rates ranging from 21% in Ghana [9] to 29% in Gabon [10] and 36% in Senegal [11], but none of these studies focus on children.

A high prevalence of Panton-Valentine Leukocidin (PVL), a cytolytic pore forming toxin, has been reported among clinical *S*. *aureus* isolates from Ghana (60%) and other West African countries [12,13]. Other toxins, such as the Toxic-shock-syndrome-toxin 1 (TSST-1) are often neglected, although remarkably prevalent in some African settings, as demonstrated in the Congo (18%) [14]. In case of autoinfection or transmission to other children those highly virulent isolates may cause severe infections in paediatric populations.

Similarly, regular surveillance of multidrug resistant (MDR) or methicillin resistant *S*. *aureus* (MRSA) is essential for clinicians, as second line therapies are often not available or contraindicated in children. Within West Africa MRSA rates from clinical samples range from 3% in Ghana to 20% in Nigeria [12,15] and a substantial number of MRSA (23%) from this region have been characterized as community-acquired MRSA (CA-MRSA) [16].

This surveillance study aims to identify demographic and season specific carriage rates, clonal types, antimicrobial susceptibilities and virulence markers of nasal *S*. *aureus* isolates collected from children living in the Ashanti Region of Ghana.

## Methods

### Isolation of bacterial strains

Study site was the Agogo Presbyterian Hospital, a district hospital with approximately 250 patient beds located in the Asante Akim North municipality in Ghana. The Asante Akim North municipal area has a population of approximately 142,400 inhabitants, spread over an area of 1,160 square kilometres. The region has a tropical climate and is mainly covered by secondary rain forest and cultivated land. The area is holoendemic for malaria transmission.

Nasal swabs from both anterior nares were taken from all children admitted to the pediatric ward between April 2014 and January 2015. Swabs were taken during the admission process, stored at 4°C and plated within a few hours on Columbia and Mannitol Salt Agar plates (all Oxoid, Basingstoke, UK) and incubated at 37°C for 24 hours. For each sample, all morphologically different colonies indicative for *S*. *aureus* were selected and phenotypically confirmed by standard gram staining and the 4–24 hour tube test for free coagulase in rabbit-citrate-plasma (Becton and Dickinson®; Heidelberg, Germany). *S*. *aureus* ATCC 33592 (MRSA; American Type Culture Collection, Wesel, Germany) and *S*. *epidermidis* DSM 20044 (German Collection of Microorganisms and cell cultures, Braunschweig, Germany) served as positive and negative controls.

The Committee on Human Research, Publications and Ethics, School of Medical Science, Kwame Nkrumah University of Science and Technology, Kumasi, Ghana provided ethical approval for this study. All participants were informed about the study’s purpose and procedures. Written informed consent was obtained from the parents or the guardian on behalf of the study children prior to study enrolment.

### Determination of antimicrobial susceptibility

All strains were analysed for antimicrobial susceptibility with the Kirby-Bauer disk diffusion method on Mueller-Hinton agar including the antibiotics cefoxitin, clindamycin, erythromycin, penicillin, linezolid, ciprofloxacin, tetracycline, trimethoprim-sulfamethoxazole, tigecycline, gentamicin, and vancomycin (Oxoid, Basingstoke, United Kingdom) according to the 2015 European Committee on Antimicrobial Susceptibility Testing (EUCAST) guidelines (www.eucast.org). MRSA isolates were additionally tested for vancomycin resistance by E-test [17]. Isolates exhibiting resistance to three or more antimicrobial classes were defined as multidrug resistant (MDR), as described before [18].

### Molecular typing of *S*. *aureus* strains and the presence of virulence factors

*S*. *aureus* isolates were discriminated by *spa*-typing as described before [19]. The *spa*-types and clonal complexes (CC) were determined using the StaphType software and the Ridom SpaServer (http://spaserver.ridom.de/). Unknown *spa*-types were uploaded and assigned to new *spa*-types by the Ridom StaphType software (http://spaserver.ridom.de/). Multi-locus-sequence-typing (MLST) was performed on all isolates with new *spa*-types and on those, which could not be assigned to a CC by the Ridom spa server. For MLST typing, the DNA sequence analyses were carried out employing the *S*. *aureus* MLST site (http://saureus.mlst.net/).

For *spa*-typing, a cell suspension was heated for 10 minutes at 95°C. Cell debris was pelleted by centrifugation (5 min, 20,000 rpm, RT). Amplification of the *spa* gene was achieved by using standard primers as previously described [19].

For MLST PCRs, chromosomal DNA was purified using the PrestoSpin D BUG Kit (Molzym, Bremen, Germany) according to the suppliers’ instructions. For cell lysis, the buffer was supplemented with 10 μl lysostaphin (5 mg/ml; Genmedics, Reutlingen, Germany). Gene amplification was executed as previously described [20].

All strains were additionally tested by PCR for the presence of the toxic shock syndrome toxin (TSST) and the *lukFS*-genes encoding both subunits of the Panton-Valentine leukocidin (PVL) [21,22]. Typing of the Staphylococcal cassette chromosome *mec* (SCC*mec*) and screening for the presence of *mecA* and *mecC* has been performed for all MRSA isolates by PCR as previously described [23,24].

In all PCR reactions the high fidelity Phusion polymerase (Finnzymes, New England Biolabs, Ipswich, USA) was used according to the suppliers’ manual. Prior to sequencing (Seqlab, Göttingen, Germany), all PCR products were purified with the GeneJET gel extraction kit (Thermo Scientific, Rochester, USA).

### Epidemiological analysis

Categorical variables were described as frequencies and percentages. Continuous variables were described using medians and their corresponding interquartile ranges (IQRs). The Student’s t-test and chi-square test were used to compare means and proportions respectively, with a p-value <0.05 being considered statistically significant. All data analyses were performed with Stata 14 (StataCorp LP, College Station, USA).

## Results

Nasal swabs from 544 children were collected during the study period. The most common causes for admission were malaria (57.7%), lower respiratory tract infections (31.1%), urinary tract infections (16.7%) and gastrointestinal infections (13.6%). The median age of patients was 2 (IQR: 1–5) years and 255 (46.9%) study children were female. In total 120 (22.1%) swabs were positive for *S*. *aureus* of which 3 revealed two different *S*. *aureus* isolates, resulting in a total of 123 *S*. *aureus* isolates. *S*. *aureus* carriage was similarly distributed (p = 0.33) among girls (n = 61/255; 23.9%) and boys (n = 59/289; 20.4%) (Table 1).

**Table 1:** Demographic characteristics of study participants.

Characteristics	Total (%)	*S*. *aureus* carrier (%)	non-*S*.*aureus* carrier (%)
	(N = 544)	(N = 120)	(N = 424)
Sex, female (%)	255 (46.9)	61 (50.8)	194 (45.8)
Age, median months (IQR)	32 (17–60)	41 (24–78)	29 (15–52)

IQR, interquartile range.

**Fig 1:**
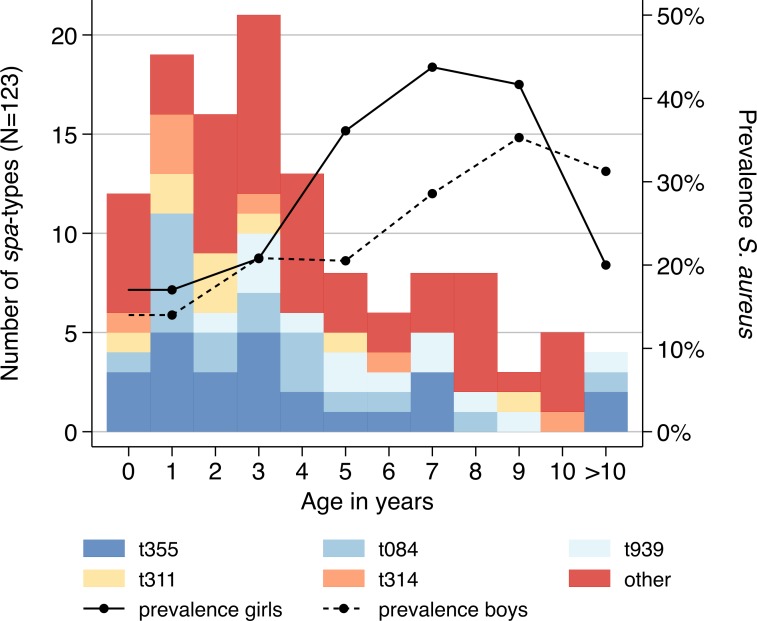
Age and sex distribution of *spa*-types. Distribution of *spa*-types (n = 123) and nasal *Staphylococcus aureus* prevalence by 2-years age-groups and sex (n = 544). Girls (continuous line) are colonized when they are approximately 2 years younger than boys (dashed line).

*S*. *aureus* carriers had a higher median age of 3 years (IQR: 2–6) compared to children without *S*. *aureus* colonization (median = 2 years; IQR = 1–4; p = 0.004). In both, girls and boys, the prevalence of *S*. *aureus* increased with age, however female patients seemed to be colonised at an earlier age (Fig 1). However, case numbers for each age group were not sufficient to perform significance testing. The highest *S*. *aureus* prevalence in girls was observed in the age-group 6–8 years (n = 7; 43.7%) and in boys in the age-group 8–10 years (n = 6; 35.2%). Above the age of 10, carriage rates dropped in both sexes. Apart from nine carriers with not further characterized skin infections, there were no indications for a link between *S*. *aureus* carriage and an underlying infection.

The 123 isolates were classified into 35 different *spa*-types and 19 sequence types (ST). The most commonly detected *spa*-types (i.e., n >5) were t355 (n = 25; 20.3%), t084 (n = 18; 14.6%), t939 (n = 13; 10.6%), t311 (n = 9; 7.3%) and t314 (n = 7; 5.7%), corresponding to ST152, ST15, ST45 and ST121 (Table 2). Two isolates were newly assigned to the two *spa*-types t15727 and t15728, which belong to ST508 and ST707, respectively. Based on *spa*-typing, the following CCs were identified: CC152 (n = 36), CC15 (n = 21), CC45 (n = 19), CC121 (n = 19), CC1 (n = 6), CC30 (n = 6), CC8 (n = 5), CC88 (n = 3), CC5 (n = 1). All *spa*-types were randomly distributed throughout age, except t939, which only occurred in children above the age of 1 year (Fig 1).

**Table 2:** Characterization of nasal *Staphylococcus aureus* isolates (n = 123).

CC	ST	*spa* types (n)	MRSA [n(%)]	MDR [n(%)]	PVL [n(%)]	TSST [n(%)]
CC1	ST1	t127 (3), t591 (1)	0 (0.0)	0 (0.0)	2 (66.7)	2 (66.7)
	ST72	t537 (2)	0 (0.0)	0 (0.0)	0 (0.0)	1 (50.0)
CC121	ST121	t311 (9), t314 (7), t645 (1), t1114 (2)	0 (0.0)	3 (15.8)	13 (68.4)	2 (10.5)
CC15	ST15	t84 (18), t085 (1), t346 (1), t385 (1)	0 (0.0)	2 (9.5)	14 (66.7)	3 (14.3)
CC152	ST152	t1096 (3), t355 (25), t1123 (2), t1172 (1), t1299 (4)	1 (2.9)	7 (20.0)	30 (85.7)	3 (8.6)
	ST377	t5047 (1)	0 (0.0)	0 (0.0)	1 (100.0)	0 (0.0)
CC30	ST30	t318 (1), t363 (3), t2147 (1)	0 (0.0)	0 (0.0)	4 (80.0)	0 (0.0)
	ST30, ST33, ST55	t21 (1)	0 (0.0)	1 (100.0)	0 (0.0)	0 (0.0)
CC45	ST45	t939 (13), t157 (1), t4454 (5)	1 (5.3)	1 (5.3)	3 (15.8)	1 (5.3)
CC508	ST508	t15727 (1), t1510 (1)	0 (0.0)	0 (0.0)	0 (0.0)	2 (100.0)
CC5	ST5	t450 (1)	0 (0.0)	1 (100.0)	0 (0.0)	0 (0.0)
CC707	ST707	t15728 (1)	0 (0.0)	0 (0.0)	0 (0.0)	1 (100.0)
CC8	ST8	t8 (3), t1476 (1)	0 (0.0)	0 (0.0)	0 (0.0)	0 (0.0)
	ST18	t451 (1)	0 (0.0)	1 (100.0)	1 (100.0)	0 (0.0)
CC88	ST88	t186 (1), t4104 (2)	0 (0.0)	0 (0.0)	3 (100.0)	0 (0.0)
singleton	ST3248	t6063 (2)	0 (0.0)	0 (0.0)	0 (0.0)	2 (100.0)
singleton	ST944	t616 (2)	0 (0.0)	0 (0.0)	0 (0.0)	0 (0.0)

CC: Clonal Complex; ST: Sequence Type; MRSA: Methicillin-resistant *Staphylococcus aureus*; MDR: Multidrug resistant; PVL: Panton-Valentine leukocidin; TSST: Toxic shock syndrome toxin.

**Fig 2:**
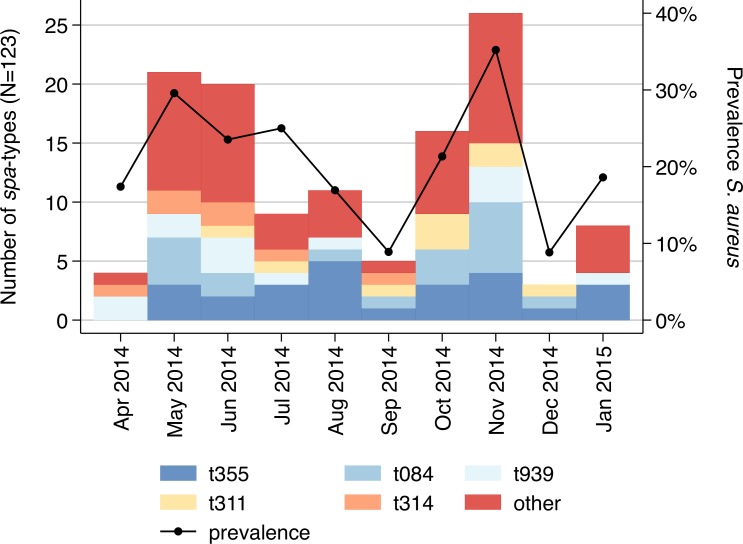
Temporal distribution of *spa*-types. Distribution of *spa*-types (n = 123) by month. The black line indicates the *Staphylococcus aureus* prevalence (n = 544).

The *S*. *aureus* prevalence revealed seasonal fluctuations in nasal carriage (Fig 2). The highest carriage rates were observed during May (n = 21; 29.6%) and November (n = 25; 35.2%), which corresponds to peaks of the two rainy seasons within the study area (p = 0.007). Notably, the frequent *spa*-types (i.e., n >5) occurred throughout the whole study period, while the more uncommon *spa*-types were primarily observed during the months of the rainy seasons.

PVL and TSST-1 were detected in 71 (57.7%) and 17 (13.8%) isolates respectively. Among all STs, which contain five or more isolates, ST152 (n = 30; 85.7%) and ST30 (n = 4; 80.0%) revealed the highest PVL positivity and ST15 the highest proportion of TSST-1 positive isolates (n = 3; 14.3%).

Two (1.6%) isolates were MRSA (SCC*mec* type IVa), classified as t1096 (ST152) and t4454 (ST45). MDR, defined as resistance to three or more different antimicrobial classes, was detected in 16 (13%) isolates. High MDR prevalences existed in ST152 (n = 7; 20.0%) and ST121 (n = 3; 15.8%). The highest resistance rates were found for penicillin (n = 117; 95.1%), tetracycline (n = 66; 53.7%) and erythromycin (n = 14; 11.4%) (Table 3). All isolates were susceptible to linezolid and vancomycin.

**Table 3:** Antibiotic resistance profile of nasal *Staphylococcus aureus* isolates (n = 123).

Antibiotic	Resistance [n (%)]	
Penicillin	117	(95.1)
Tetracycline	66	(53.7)
Erythromycin	14	(11.4)
Clindamycin	7	(5.7)
Trimethoprim-sulfamethoxazole	4	(3.3)
Gentamicin	3	(2.4)
Cefoxitin	2	(1.6)
Ciproflocacin	2	(1.6)
Linezolid	0	(0.0)
Tigecycline	0	(0.0)
Vancomycin	0	(0.0)
Multidrug resistance^§^	16	(13.0)

^§^ Resistance to three or more antimicrobial classes.

## Discussion

The present study identifies an age- (p = 0.004) and season-dependent (p = 0.007) nasal *S*. *aureus* colonisation and genotype pattern among children living in rural Ghana. Similar age-dependent parabolic carriage rates have been described in the Netherlands, however peak incidences occurred at a slightly older age (10 years) [5]. Earlier colonisation after the first year of life might be caused by different exposures in rural Ghana compared to industrialized countries. For instance large family sizes and lower socio-economic development have been associated with *S*. *aureus* carriage [7,25]. However, the total rate of *S*. *aureus* carriers in the present study (22.1%) tends to be lower than in industrialized countries, such as the United States (36.9%; age-group 1–19 years) and the Netherlands (36,0%; age-group 1–19 years) [5,26]. Children aged 2–15 years from The Gambia revealed a carriage rate of 27.3% [27]. Other carriage studies from West Africa do not allow a comparison, as either children were not included or rates were not stratified by age-groups [9,11,28].

Differences might also be attributed to the variable nasal carriage of *Streptococcus pneumoniae* and *Haemophilus influenzae*, which previously showed a negative correlation for co-colonisation with *S*. *aureus* [5,8,29,30]. This association has been discussed in the context of lower *S*. *aureus* carriage rates among underprivileged populations, such as indigenous Australians, Hispanic Americans and Bedouin Israelis compared to the rest of the population [8,26,30].

Co-colonisation might play a role in rural Ghana, in particular for the observed seasonal distribution, with prevalence peaks during the two rainy seasons, which occur from May to June and from October to November (http://www.meteo.gov.gh). In Israel the highest *S*. *aureus* prevalence was observed during summer, when *S*. *pneumoniae* and *H*. *influenzae* carriage were the lowest [8]. In Sweden and the United Kingdom prevalence rates culminated in spring and autumn, respectively, when viral respiratory infections are on the rise [31,32]. Similarly, an increase of enveloped respiratory viruses occurs during the rainy seasons in Ghana [33]. To our knowledge no seasonality data for *S*. *aureus* colonisation is yet available for West Africa.

This study reports equal *S*. *aureus* carriage rates for boys and girls, the latter colonized when they are approximately two years younger than male study participants. Interestingly, male sex has been repeatedly identified as a risk factor for carriage in industrialized countries [5,8,26], while most studies from sub-Sahara Africa did not observe any difference [9,10,27].

The two most frequently detected *spa*-types, t355 and t084, have been described before as the major *S*. *aureus* lineages in asymptomatic carriers as well as in clinical isolates from Ghana [9,12]. Indeed, those *spa*-types have always been among the most frequently detected in nasal carriage studies throughout West Africa [11,28]. *Spa*-type t939, with a prevalence of 11% being the third most common in this study, has only been detected sporadically in other West and Central African countries, such as Angola, Gabon, Nigeria and São Tomé and Príncipe [34–37]. In Ghana, t939 isolates have been only reported once from a nasal carrier and from the wounds of two patients with Buruli ulcer [9,38]. In the past *spa*-type t939 has been associated with transmission among pig farms in the Netherlands [39]. Livestock associated transmission might be one explanation for the exclusive detection of this *spa*-type in children above one year in the present study.

The MRSA prevalence among nasal carriers is below two percent, which is similar to what has been previously described in Ghana [9,12]. The detected MRSA spa-types t1096 and t4454 belong to CC152 and CC45 respectively, which are well-described MRSA CCs, circulating on the African continent [40]. However, the *spa*-type t1096 has so far only been detected in a methicillin susceptible *S*. *aureus* (MSSA) isolate in Ghana whereas a methicillin resistant t4454 *S*. *aureus* has not been described before in Africa. Compared to a nasal carriage study from Ghana, conducted between 2011 and 2012, MDR rates seem to increase from 6% reported previously to 13% detected in the present study [9]. Similarly, penicillin and erythromycin resistance rate increase from 91% to 95% and from 2% to 11%, respectively [9]. These two studies have been conducted in different areas; therefore those trends must be interpreted cautiously. A high tetracycline resistance has been previously found in a rural community (50%) in Ghana when compared to an urban setting (18%) [9]. The authors suggested the common use of tetracycline on Ghanaian livestock farms to be correlated to this finding.

PVL is highly prevalent (58%) among the collected isolates, similar to those from a remote Gabonese Pigmy population (56%) [41]. Other carriage studies from sub-Sahara Africa reported a lower PVL prevalence ranging from 8% in Angola to 36% in São Tomé and Príncipe [9,11,36,37]. Even lower PVL rates have been reported from the United States with 1% among MSSA [42]. The observed high number of PVL-positive MSSA in combination with an increased risk of human-to-human transmission due to poor sanitary conditions and overcrowding in rural Ghana could pose a serious threat for the generation of PVL-positive MRSA clones through horizontal gene transfer.

The study presented here has a few limitations. The recruitment was hospital-based and only children admitted to the paediatric ward were included into the study, which may therefore not be representative for the local healthy community. Antibiotic consumption, previous hospital stays and other risk factors, which may explain the increasing MDR rates, have not been assessed in this study. The cross-sectional design of the study with a single nasal culture does not allow the classification of individuals as persistent or intermittent carriers. This distinction may be relevant, as persistent carriers have a higher risk to acquire *S*. *aureus* infections and patients with a negative nasal culture might actually be intermittent carriers.

## Conclusion

The results of the present study suggest that nasal *S*. *aureus* carriage among children in Ghana is dependent on age and seasonality, with children above one year being colonized at a younger age compared to previous studies from industrialized countries. To what extent socioeconomic conditions, climatic factors or co-colonization with other pathogens play a role for nasal *S*. *aureus* carriage in this geographic region needs to be investigated in further studies. High PVL rates can be considered a serious threat for the development of virulent MRSA in the future. Already today, physicians must be aware of increasing antibiotic resistance among paediatric *S*. *aureus* isolates when comparing to previous Ghanaian studies.

## Supporting Information

S1 Dataset Complete dataset for the analysis. (TXT)
